# Preparation and Properties of Stereocomplex of Poly(lactic acid) and Its Amphiphilic Copolymers Containing Glucose Groups

**DOI:** 10.3390/polym12040760

**Published:** 2020-03-31

**Authors:** Liyan Qi, Qianjin Zhu, Dan Cao, Tingting Liu, Kevin R Zhu, Kaixin Chang, Qinwei Gao

**Affiliations:** College of Chemical Engineering, Nanjing Forestry University, Nanjing 210037, China; 18362985223@163.com (L.Q.); 18260077835@163.com (Q.Z.); cd15861810289@163.com (D.C.); liu1103746992@163.com (T.L.); kevinrzhu123@gmail.com (K.R.Z.); ckx851127@gmail.com (K.C.)

**Keywords:** stereocomplex, amphiphilic polymer, poly(l-lactic acid), poly(d-lactic acid-*co*-glucose) copolymer, glucose

## Abstract

The stereocomplex of poly(lactic acid) containing glucose groups (sc-PLAG) was prepared by solution blending from equal amounts of poly(l-lactic acid) (PLLA) and poly(d-lactic acid-*co*-glucose) (PDLAG), which were synthesized from l- and d-lactic acid and glucose by melt polycondensation. The methods, including ^1^H nuclear magnetic resonance spectroscopy (^1^H NMR), gel permeation chromatography (GPC), differential scanning calorimetry (DSC), X-ray diffraction (XRD), fourier transform infrared spectroscopy (FT-IR), thermogravimetric analysis (TGA), polarizing microscope (POM), scanning electron microscope (SEM), transmission electron microscope (TEM), and contact angle were used to determine the effects of the stereocomplexation of enantiomeric poly(lactic acid) (PLA) units, the amphiphilicity due to glucose residues and lactic acid units, and the interaction of glucose residues with lactic units on the crystallization performance, hydrophilicity, thermal stability, and morphology of samples. The results showed PDLAG was multi-armed, and partial OH groups of glucose residues in PDLAG might remain unreacted. The molecular weight (*M*_w_), dispersity (*Ɖ*), and glucose proportion in the chain of PDLAG thereby had significant effects on sc-PLAG. There were the stereocomplexation of enantiomeric lactic units and the amphiphilic self-assembly of PDLAG in sc-PLAG, which resulted in glucose groups mainly in the surface phase and lactic units in the bulk phase. The sc-PLAG only possessed the stereocomplex crystal owing to the interaction between nearly equimolar of l-lactic units of PLLA and d-lactic units of PDLAG, and had no homo-crystallites of l- or d-lactic units, which improved the melting temperature (*T*_m_) of sc-PLAG about 50 °C higher than that of PLLA. Glucose groups in sc-PLAG played an important role by forming heterogeneous nucleation, promoting amphiphilic self-assembly, and affecting the ordered arrangement of lactic units. The glass transition temperature (*T*_g_), the melting temperature (*T*_m_), crystallinity, crystallization rate, and water absorption of sc-PLAG showed similar changes with the increased glucose content in feeding. All these parameters increased at first, and the maximum appeared as glucose content in feeding about 2%, such as the maximum crystallinity of 48.8% and the maximum water absorption ratio being 11.7%. When glucose content in feeding continued increasing, all these performances showed a downward trend due to the decrease of arrangement regularity of lactic acid chains caused by glucose groups. Moreover, the contact angle of sc-PLAG decreased gradually with the increased glucose content in feeding to obtain the minimum 77.5° as the glucose content in feeding being 5%, while that of PLLA was 85.0°. The sc-PLAG possessed a regular microsphere structure, and its microspheres with a diameter of about 200 nm could be observed. In conclusion, sc-PLAG containing proper glucose amount could effectively enhance the crystallinity, hydrophilicity, and thermal stability of PLA material, which is useful for drug delivery, a scaffold for tissue engineering, and other applications of biomedicine.

## 1. Introduction

Poly(lactic acid) (PLA), as an environmentally friendly thermoplastic polyester with excellent reproducibility of raw materials, good biocompatibility, biodegradability [1,2,3,4], and nontoxicity to the human body [5,6], is widely used in tissue engineering, drug controlled release, and packaging materials [7,8,9,10], whereas its poor thermal stability, slow crystallization rate, high crystallinity, and poor hydrophilicity confine its application scope [11,12,13]. To overcome the mentioned shortcomings, the performance of PLA materials can be improved through physical blending [14,15], copolymerization [16,17,18], the addition of a nucleation agent [19], and preparation of nanoparticle [20,21,22].

In order to improve the thermal properties of PLA materials, the two optical isomers of PLA, poly(l-lactic acid) (PLLA), and poly(d-lactic acid) (PDLA) are usually mixed to prepare the stereocomplex of poly(lactic acid) (sc-PLA). The sc-PLA with stereocomplex crystal structure may significantly boost the thermal stability of PLA, and its melting temperature (*T*_m_) is about 50 °C higher than that of the neat PLLA [8,23]. Moreover, because PLLA with high molecular weight (*M*_w_) and PDLA with high *M*_w_ usually cannot mix well, they cannot completely form the stereocomplex crystal structure (SC), and some homo-crystallites (HC) may form meanwhile. PLA grafted with cellulose could result in low melting temperature, good biocompatibility [24], and less hydrophobicity [10,25]. Glucose, as the most common monosaccharide in nature, is well-known for its extensive sources, low price, nontoxicity, and high hydrophilicity, etc. Glucose possessing five hydroxyl groups is easily modified with PLA by blending [14,26] or copolymerization [27]. Because of its advantages, such as low cost, simple technology, and convenient operation, the direct polycondensation may be used to prepare PLA and its copolymers, and the obtained PLA usually has low molecular weights [28]. Luo et al. [29] used glucose (Glu) and l-lactic acid (LLA) as starting material to synthesize poly(l-lactic acid-*co*-glucose) via melt polymerization at different molar feed ratios (n(l-LA ):n(Glu) being more than 50:1). The obtained copolymer was multi-armed with LLA chains, but the number and the length of LLA arms were not discussed clearly. The *M*_w_ of all copolymers was less than 5900 Da, and all dispersity (*Ɖ*) values *(M*_w_/*M*_n_) were less than 2. PLA with low *M*_w_ is liable to copolymerize with sugars, while it or its copolymers are easy to form perfect SC structure when blended. Cao et al. [30] synthesized two kinds of copolymers, such as poly(l-lactic acid-*co*-glucose) (PLLAG) and poly(d-lactic acid-*co*-glucose) (PDLAG), by melt polymerization with d-lactic acid, LLA, and glucose as raw materials. The *M*_w_ of PLLAG and PDLAG was about 15,000–20,600 with *Ɖ* value less than 2. Then, the stereocomplex of PLLAG and PDLAG was prepared by solution blending method. The ratios of PLLAG to PDLAG and glucose content in feeding had significant impacts on stereocomplex properties. These modifications of PLA are conducive to the performance improvement of PLA materials, which is one focus of contemporary green chemistry [31,32].

In this paper, we discussed the melt copolycondensation of lactic acid with glucose and the stereocomplex of PLLAG and PDLAG, in the light of our previous study [30]. We attempted to improve the comprehensive performances of PLLA materials by modifying linear PLLA homopolymer with multi-armed PDLAG through stereocomplexation of enantiomeric poly(lactic acid) units and the effect of amphiphilicity due to glucose residues and lactic acid units. PDLAG was prepared from d-lactic acid and glucose by melt polycondensation, while PLLA was prepared from l-lactic acid. Then, PLLA and PDLAG were mixed by solution blending to obtain the stereocomplex of PLLA and PDLAG (sc-PLAG—stereocomplex of poly(lactic acid) containing glucose groups). The glucose groups existing in the stereocomplex sc-PLAG could improve the hydrophilic performance of sc-PLAG. The effect of glucose groups on the preparation of sc-PLAG and the influence of glucose content in feeding on sc-PLAG were investigated so as to enhance the thermal stability, crystallization, and hydrophilic performance of poly(lactic acid) materials.

## 2. Materials and Methods

### 2.1. Materials

l-lactic acid and d-lactic acid (AR, 90%, MUSASHINO CHEMICAL (CHINA) CO., LTD. Yichun, China), anhydrous glucose (AR, Sinopharm Chemical Reagent Co., Ltd. Shanghai, China), stannous chloride (SnCl_2_) (AR, Shanghai Jiuyi Chemical Reagent Co., Ltd. Shanghai, China), p-toluenesulfonic acid (TSA) (AR, Shanghai Lingfeng Chemical Reagent Co., Ltd. Shanghai, China), methanol (AR, Nanjing Chemical Reagent Co., Ltd. Nanjing, China), and trichloromethane (AR, Shanghai Pilot Chemical Corporation, Shanghai, China) were all analytical reagents available in the market.

### 2.2. Characterization Methods

DRX2500 ^1^H-NMR (Bruker Byerspin, Flanders, Switzerland) was used to record the ^1^H-NMR spectrum of PDLAG with CDCl_3_ as the solvent and tetramethylsilane (TMS) as the internal standard, and the chemical shift of chloroform (δ) was 7.26 as a reference. FT-ITIR-360 Infrared Spectrometer (Thermo Nicolet Corporation, Beijing, China) was used to determine the infrared spectra of samples, KBr tabletting, scanning range being from 400 to 4500 cm^−1^. Agilent 1100 gel permeation chromatography (Agilent Technologies (China) Co., Ltd. Beijing, China) was utilized to measure the molecular weight (*M*_w_) of PLLA and PDLAG, and tetrahydrofuran (THF) was used as the mobile phase to dissolve the sample in THF at a concentration of 1 mg/mL with a flow rate of 1 mL/min and an injection volume of 20 μL. The thermal performance of samples was measured by the DSC-200F3 differential scanning calorimeter (NETZSCH-Gerätebau GmbH, Selb, Germany). The test temperature range was 10–250 °C under the protection of N_2_ airflow, and the heating rate was 10 °C/ min. The crystallinity was calculated by Proteus Analysis software (NETZSCH-Gerätebau GmbH, Selb, Germany). X-ray diffraction (XRD) analysis was carried out on a Rigaku D/max-Ra X-ray diffractometer (Riaku Corporation, Tokyo, Japan) with Cu-Kα radiation (λ = 0.154 nm), 40 kV voltage, and electric current 30 mA. The range of 2θ was from 5° to 40° at a scan rate of 5°/min. Q5000 thermogravimetric analyzer was used to determine the TGA curve of polymers with the temperature range from 20 to 600 °C at the heating rate of 10 °C/min and the flow rate of N_2_ of 10 mL/min. The contact angles were characterized by the FACECA-D contact angle meter (Japan Concorde co., LTD, Tokyo, Japan). The granular samples were melted and compressed to be molded into a film for the characterization. The measurement time of each point was 10 s, and the contact angle was the average value of five measurements. Transmission electron microscope (TEM) images were recorded by a JEM-1400 transmission electron microscope (Nippon electronics co., LTD, Tokyo, Japan). The sample was dispersed in ethanol solvent, treated by ultrasonic for 10 min. Then, a 1 μL sample solution drawn by a sampler was added to the copper mesh containing a carbon layer and dried at room temperature. The crystal morphology was investigated by the ECLIPSE polarizing microscope (Nikon Corporation, Tokyo, Japan). The morphology of sc-PLAG powders and sections was observed by Quanta 200 scanning electron microscope (SEM) (USA FEI, Hillsboro, Oregon, USA). The granular samples were melted and compressed to be molded into the film, and then the obtained films were soaked into distilled water for 24 h at 37 ± 0.5 °C. The added weight of sheet samples was weighed and divided by the weight of dried samples to get the water absorption ratio.

### 2.3. Synthesis of Poly(l-lactic acid)

**Scheme 1:**
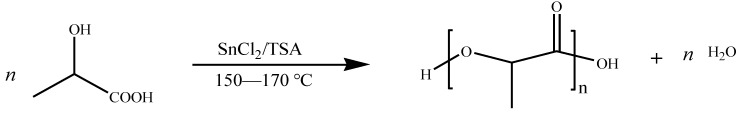
The synthetic route of poly(l-lactic acid) (PLLA).

A certain mass of l-lactic acid was weighed and added to a three-necked flask, which was heated up to 150 °C under normal pressure for 1 h, then decompressed to −0.08 MPa and reacted under this pressure for 4 h. Then, the catalyst containing stannous chloride and *p*-toluenesulfonic acid (the molar ratio of the two compounds being 1:1) was added to the flask. The catalyst amount was 0.5 wt% of the total mass of l-lactic acid. The reactor was heated up to 170 °C, and the reaction continued under a complete vacuum for 8 h. Then, the product was dissolved in trichloromethane and precipitated by methanol excess. The separated precipitates were dried in a vacuum for 10 h at 50 °C to obtain PLLA samples. The synthetic route of PLLA is shown in Scheme 1.

### 2.4. Synthesis of Poly(d-lactic acid-co-glucose) Copolymer

**Scheme 2:**
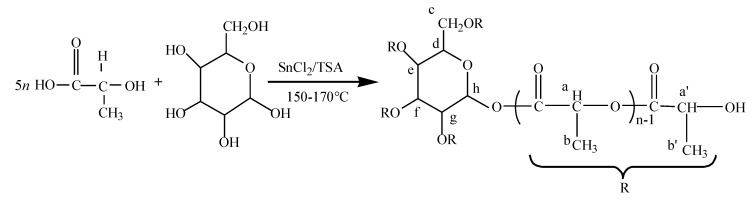
The synthetic route of poly(d-lactic acid-*co*-glucose) (PDLAG).

A certain amount of glucose and d-lactic acid was weighed and added to a three-necked flask, which was heated up to 150 °C under normal pressure for 1 h, then decompressed to −0.08 MPa and reacted under this pressure for 4 h. Then, the catalyst containing SnCl_2_ and TSA (the molar ratio of the two compounds being 1:1) was added to the flask. The catalyst amount was 0.5 wt% of the total mass of d-lactic acid and glucose. The reactor was heated up to 170 °C, and the reaction continued under a complete vacuum for 6–8 h. Then, the product was dissolved in trichloromethane and precipitated by methanol excess. The separated precipitates were dried in vacuum at 50 °C for 10 h to obtain poly(d-lactic acid-*co*-glucose) copolymer (PDLAG). The glucose content in the feeding of PDLAG samples was correspondingly expressed by the mass percentage of glucose to d-lactic acid in raw materials. So, the copolymer PDLAG obtained when the mass percentage of glucose in raw materials was 5% was named as PDLAG-5%. The other PDLAG samples were labeled with the same method. The glucose content in the feeding of the PDLA sample was 0%. The synthetic route of PDLAG is shown in Scheme 2.

### 2.5. Preparation of Stereocomplex of Poly(lactic acid) with Glucose Groups

PLLA and PDLAG with different glucose content in feeding were dissolved in trichloromethane, respectively, to prepare solutions with a 10% mass fraction. After evenly mixing equal amounts of PLLA and PDLAG solutions at room temperature, the mixture solution removed solvent by heating in the water bath at 50 °C for 10 h under atmospheric pressure and then dried at 50 °C under vacuum for 10 h to obtain the stereocomplex of poly(lactic acid) containing glucose groups, namely sc-PLAG.

## 3. Results and Discussion

### 3.1. Characterization of PDLAG

**Figure 1:**
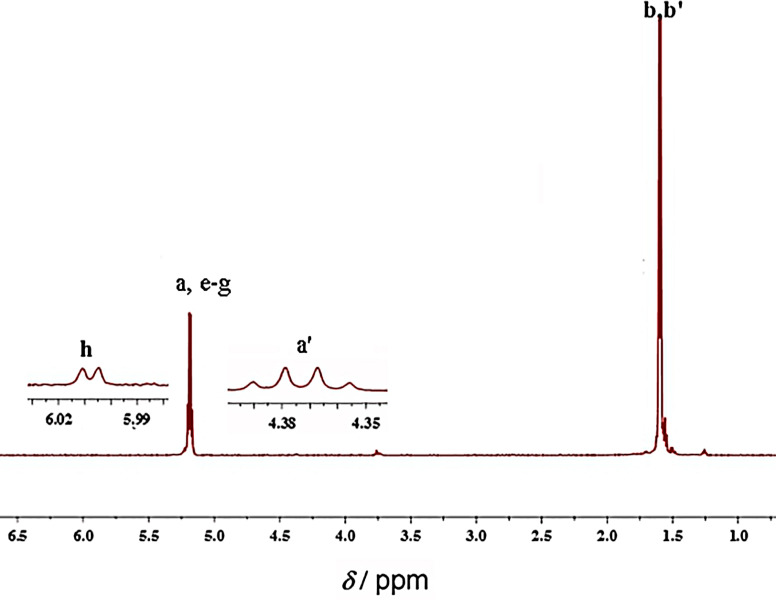
^1^H-NMR spectrum of poly(d-lactic acid-*co*-glucose) (PDLAG)-5%.

Poly(d-lactic acid-*co*-glucose) with glucose content in the feeding of 5 wt% was used as an example to perform the structural characterization of PDLAGs by FTIR and ^1^H-NMR (Figure 1). The FTIR curve showed the strong absorption of ester carbonyl at 1750 cm^−1^ and the characteristic absorptions of glucose group at 870 and 755 cm^−1^. The ^1^H-NMR spectrum showed -CH_2_ from glycosyl (H_c_, Scheme 2) at 4.27–4.34 ppm and –CH from glycosyl (H_h_, Scheme 2) at 5.96 ppm. ^1^H-NMR also showed -CH from repeat PDLA units (H_a_, Scheme 2) at 5.15–5.22 ppm and –CH from the DLA end group (H_a’_, Scheme 2) at 4.35–4.40 ppm. The ratio of the integral area of H_a’_ to that of glycosyl H_h_ could be calculated to stand for the number of grafted PDLA chains on a glucose group. The ratio of H_a’_ to H_h_ was more than 4, which meant that there were more than four PDLA chains linked to glucose residue. Namely, PDLAG could be multi-armed other than linear, and partial OH groups of glucose in PDLAG could be unreacted. The glycosyl with unreacted OH, together with the OH end group in a short DLA unit linked to it, was hydrophilic, and DLA chains were hydrophobic. So, PDLAG was amphiphilic. The results of FTIR and ^1^H-NMR indicated that the obtained PDLAGs were copolymers, and the glycosyl units were incorporated in PDLA backbones.

The *M*_n_, *M*_w_, and *Ɖ* of PLLA and PDLAG were measured by GPC, and the results are shown in Table 1. All GPC flow curves of PDLAG samples had only a single symmetrical peak, and all *Ɖ* values were less than 2, which indicated that the melt polycondensation of d-lactic acid and glucose indeed only gave the copolymer. The molecular weights of PDLAG were similar to that of PLLA, but *Ɖ* values of PDLAG were less than that of PLLA. With increasing glucose content in feeding, the molecular weights of PDLAG decreased first, reached to the lowest as glucose content in feeding being 2% (i.e., PDLAG-2%), and then increased gradually. Moreover, the *Ɖ* values of PDLAG increased with increasing glucose content in feeding. The molecular weight and *Ɖ* of PDLAG-5% were the highest. PDLAG-2% with lowest *M*_w_ and *Ɖ* about 1.90 could possess shorter chains, which could move easier when blending with PLLA. Moreover, the glucose proportion in PDLAG depended on the *M*_w_ of PDLAG, and PDLAG with high *M*_w_ had a low glucose proportion. The glucose proportion in PDLAG was calculated by the ratio of the *M*_w_ of glucose units to the *M*_w_ of the corresponding PDLAG and are listed in Table 1. With increasing glucose contents in feeding, the *M*_w_ of PDLAG might lessen, but the *Ɖ* values increased. Thus, PDLAG-2% had the lowest *M*_w_, leading to the highest glucose proportion in the chain. As glucose contents in feeding continued increasing, the percentage of low-*M*_w_ PDLAG would rise. Moreover, the fairly low-*M*_w_ PDLAG fractions could dissolve in the mixture of trichloromethane and methanol and be removed from the products. The high-*M*_w_ PDLAG fractions were left during purification. Thus, the *M*_w_ of PDLAG-4% and PDLAG-5% gradually increased, but their glucose proportions were lessened, even less than the glucose content in feeding. Moreover, PDLAG-5% possessed the highest *M*_w_, highest *Ɖ* values, and the lowest glucose proportion in the PDLAG chain.

### 3.2. DSC Analysis of sc-PLAG Thermal Properties and Crystal Structure

**Figure 2:**
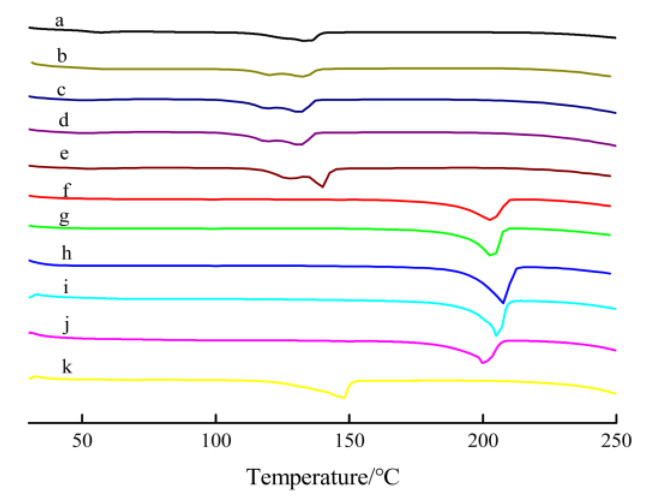
DSC curves of poly(l-lactic acid) (PLLA), PDLAG, and stereocomplex of poly(lactic acid) containing glucose groups (sc-PLAG) samples with different glucose content in feeding, a. PDLAG-5%; b. PDLAG-4%; c. PDLAG-2%; d. PDLAG-1%; e. PDLAG-0.5%; f. sc-PLAG-5%; g. sc-PLAG-4%; h. sc-PLAG-2%; i. sc-PLAG-1%; j. sc-PLAG-0.5%; k. PLLA.

In order to analyze the effects of stereocomplexation and amphiphilicity on sc-PLAG, the DSC method was used to investigate the thermal properties and crystal structure of sc-PLAG [33]. All sc-PLAG samples were prepared by mixing the equal mass ratio of PLLA and PDLAG with different glucose content in feeding. The sc-PLAG sample prepared from PLLA and PDLAG-5% was named as sc-PLAG-5%, i.e., its glucose content in feeding was 5%. The other sc-PLAG samples were labeled with the same method. Because the contents of glucose residues in PDLAG were very low, l-lactic acid units in PLLA could be considered to be equimolar to d-lactic acid units in PDLAG regardless of glucose groups in sc-PLAG. Figure 2 shows the DSC curves of PLLA, PDLAG, and sc-PLAG samples with different glucose content in feeding. The crystallinity of both PLLA and PDLAG samples was calculated by the percentage of sample melting enthalpy and the melting enthalpy of PLA with the crystallinity of 100% (93.6 J/g). The crystallinity of sc-PLAG was calculated by the percentage of sample melting enthalpy and the melting enthalpy of sc-PLA with the crystallinity of 100% (142 J/g) [33,34]. The obtained transition temperature (*T*_g_), *T*_m_, and crystallinity are summarized in Table 2. The data of other PDLAG samples by DSC are also listed in Table 2 in order to compare with sc-PLAG. According to Figure 2 and Table 2, PLLA had the *T*_m_ of 147.9 °C due to HC formed by l-lactic units [35], while PDLAG-5% also could form HC formed by d-lactic units, and its *T*_m_ was 133.1 °C. Compared with PLLA, the *T*_g_, *T*_m_, and crystallinity of PDLAG significantly decreased, indicating that glucose groups in PDLAG chains weakened the thermal stability and crystallization performance of d-lactic units. The interaction between glucose residues and d-lactic acid units would result in glucose residues involved in crystallization, decreasing the crystal perfection and crystallinity of PDLAG. The glucose residues leading to the multi-armed structure of PDLAG and more end groups in PDLAG would reduce chain regularity and decrease the crystal perfection and crystallinity of PDLAG. Moreover, low *Ɖ* and high molecular weight of PDLAG would be conducive to crystallization. Thus, less glucose proportion in the chain, high *M*_w_, and good chain regularity of PDLAG would increase the *T*_m_ of PDLAG samples. Because of the comprehensive effect of these factors, the *T*_m_ and crystallinity of PDLAG were less than those of PLLA and varied with increasing glucose content in feeding. That is the glucose content in feeding affected the molecular weights, *Ɖ* values of PDLAG, and glucose proportions in PDLAG. PDLAG-2% had the smallest *T*_m_, mainly owing to its lowest *M*_w_ and highest glucose proportion in PDLAG. On the other hand, PDLAG-0.5% had the highest *T*_m,_ mainly due to its lowest *Ɖ* along with relatively high *M*_w_, and the lowest glucose proportion in PDLAG.

When equimolar PLLA and PDLAG with different glucose content in feeding were blended to obtain sc-PLAG, all sc-PLAG samples only had one *T*_m_ more than 200 °C and 50 °C higher than that of PLLA. The *T*_m_ of sc-PLAG corresponded to the *T*_m_ of SC of PLA [35], which showed that glucose groups of PDLAG could not block the formation of SC structure of sc-PLAG. All sc-PLAG samples only formed SC structure instead of HC structure, indicating that SC structure was superior to HC in sc-PLAG [33]. The preforming stereocomplex crystallite might act as nucleating agents in the blending process and significantly increase the crystallization rate of PLA [35,36]. We could also find from Table 2 that the melting point of sc-PLAG varied regularly with glucose content in feeding. The *T*_m_ of sc-PLAG-0.5% was 205.6 °C, and then the *T_m_* of sc-PLAG increased with the increased glucose content in feeding to reach to the maximum of 208.1 °C as the glucose content in feeding being 2%. Meanwhile, it could be observed from Table 2 that the effect of glucose content in feeding on the crystallinity of sc-PLAG was similar to its effect on the *T*_m_ of sc-PLAG. The crystallinity of PDLAG-5% and PLLA was 21.5% and 48.4%, respectively, while the crystallinity of each sc-PLAG was more than that of PDLAG-5% and less than or equal to that of PLLA, indicating that the interaction between PLLA and PDLAG improved the crystallization capacity of PLA. The crystallinity of sc-PLAG-0.5% was 35.9%, the crystallinity of sc-PLAG gradually increased with the increase of glucose content in feeding up to the maximum 48.8% as glucose content in feeding being 2%. However, when glucose content in feeding continued increasing, the crystallinity of sc-PLAG gradually decreased to 38.3% of sc-PLAG-5%. The influence factors of the crystallization of sc-PLAG might include stereocomplexation of enantiomeric PLA units, the amphiphilicity due to glucose residues and lactic acid units, and the interaction between glucose residues and l-lactic and d-lactic acid units. In sc-PLAG, the glucose groups interacted with lactic acid units to a certain extent, which might lead to the less ordered arrangement of l-lactic and/or d-lactic chains, the increase of crystal defects, and the decreasing amounts of SC, so that both *T*_m_ and crystallinity of sc-PLAG decreased. In PDLAG, the glycosyl with unreacted OH group of glucose and grafted likely by short DLA units with OH end groups was hydrophilic, and DLA chains were hydrophobic. The amphiphilic PDLAG could bring forth self-assembly and promote the aggregation of lactic units, which might improve the ordered arrangement of lactic acid chains, decrease crystal defects, and increase the amounts of SC. Therefore, the *T*_m_ and crystallinity of sc-PLAG increased. The stereocomplexation, amphiphilicity, and interaction in sc-PLAG were related to the motion of PDLAG affected by the chain structure and molecular weight of PDLAG. Multi-arm PDLAG with low *M*_w_ and short branches could move easily to form SC structure with linear PLLA regardless of the effects of PLLA. Moreover, the PDLAG sample with low *M*_w_ had high glucose proportion in molecular chains, which indicated high glucose amount in its sc-PLAG and increased amphiphilic interaction. The comprehensive effects of these factors resulted in different trends in the *T*_m_ and crystallinity of sc-PLAG with the change of glucose content in feeding. When a small amount of glucose group existed in sc-PLAG, the self-assembly of amphiphilic groups was weak, and the interaction between glucose groups and lactic units could reduce the ordered arrangement and the amount of SC, which decreased the *T*_m_ and crystallinity of sc-PLAG. The amphiphilic interaction of PDLAG gradually strengthened with increasing glucose content in feeding, and the ordered arrangement of lactic acid chain increased, which would improve the crystallization of sc-PLAG so that the *T*_m_ and crystallinity of sc-PLAG increased. When glucose content in feeding was 2%, the glucose amount in sc-PDLAG-2% was the biggest. The interaction between glucose groups and lactic units and the amphiphilic interaction achieved balance; thus, the *T*_m_ and crystallinity of sc-PLAG reached to the maximum. As glucose content in feeding continued to increase, the *M*_w_ of PDLAG increased gradually, so the glucose amount in sc-PDLAG decreased, and the motion of PDLAG was reduced. The interaction between the glucose groups and lactic units gradually enhanced, and the amphiphilic effect weakened, which resulted in the decrease of SC perfection and crystallinity, namely, the *T*_m_ and crystallinity of sc-PLAG decreased.

The *T*_g_ of PLLA, PDLAG, and sc-PLAG in Table 2 showed a similar trend like *T*_m_ with the change of glucose content in feeding. It’s well known that both *M*_w_ and *Ɖ* have an obvious influence on the *T*_g_ of polymers. PDLAG-0.5% with relatively high *M*_w_ and small *Ɖ* had the highest *T*_g_ (60.6 °C), while the *T*_g_ of PDLAG-5% with highest *M*_w_ and relatively big *Ɖ* was 52.9 °C. The *T*_g_ of all PDLAG samples was less than that of PLLA (with *T*_g_ being 65.7 °C), which showed that the *T*_g_ was lowered by the introduction of a small mass of glucose groups. The *T*_g_ of sc-PLAG-0.5% (being 53.9 °C) was less than that of PLLA. With increasing glucose contents in feeding, the *T*_g_ of sc-PLAG-1% and sc-PLAG-2% increased and reached the maximum *T*_g_ similar to that of PLLA. If glucose content in feeding continued to increase, the *T*_g_ of sc-PLAG gradually decreased to the smallest *T*_g_ of sc-PLAG-5%. The influence factors of *T*_g_ might include the *M*_w_ and *Ɖ* of PDLAG, the steric effect of glucose groups, crystallinity, the interaction of glucose groups with lactic units, etc., which would vary with glucose content in feeding. The comprehensive effects of these factors might change the *T*_g_ of sc-PLAG with glucose content in feeding.

In conclusion, the SC structure and glucose content in feeding made a significant impact on the *T*_g_, *T*_m_, and crystallinity of sc-PLAG. Thus, the preparation of the stereocomplex and the adjustment of glucose content in feeding could improve the mechanical property and thermal stability of PLA materials [35].

### 3.3. XRD Analysis of sc-PLGA Crystal Structure

**Figure 3:**
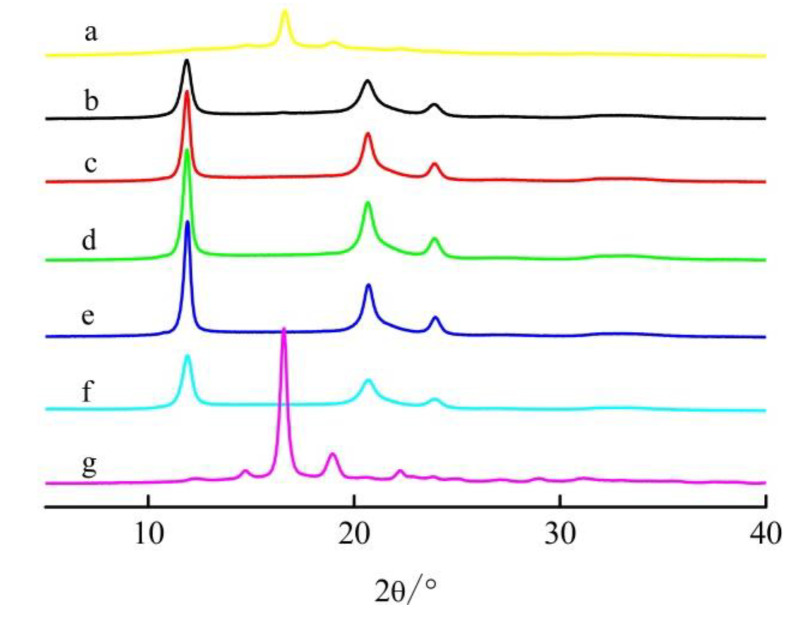
XRD curves of PLLA, PDLAG-5%, and sc-PLAG samples with different glucose content in feeding; the glucose content in the feeding of samples: a. PDLAG-5%; b. 5%; c. 4%; d. 2%; e. 1%; f. 0.5%; g. PLLA.

Figure 3 shows the XRD curves of PLLA, PDLAG-5%, and sc-PLAG samples with different glucose content in feeding. The XRD curve of PLLA presented four diffraction peaks at 2θ = 14.8°, 16.6°, 19.0°, and 22.2°, corresponding to the reflection of (010), (200)/(110), (203), and (015) planes of PLA α-form crystal, respectively [37], which could be assigned to the HC diffraction peaks of lactic units, i.e., PLLA formed HC. At the same time, we also found two diffraction peaks at 2θ = 16.6° and 19.0° in PDLAG-5%, but the peak intensity was significantly weaker than those of PLLA, indicating that the addition of glucose could reduce the ordered arrangement of lactic units and thus increase the crystal defects, which was consistent with the above DSC conclusions.

Moreover, the sc-PLAG curves all presented diffraction peaks at 2θ = 11.9°, 20.7°, and 23.9°, corresponding to diffractions of (110), (300/030), and (220) planes of stereocomplex crystal, respectively, which are the characteristic peaks of SC [36]. These results indicated that d-lactic units in PDLAG and PLLA could form SC through stereocomplexation [37]. Figure 3 shows that all sc-PLAG only possessed SC diffraction peaks and no HC diffraction peaks, which meant that only SC existed in sc-PLAG, namely, SC could be formed more easily than HC [33]. Moreover, the 2θ of diffraction peaks of sc-PLAG almost didn’t change with glucose content in feeding, which suggested that glucose was less involved in the SC of PLA and did not affect the crystal form of sc-PLAG. When glucose content in feeding was 0.5%, the intensity of the sc-PLAG-0.5% diffraction peak was the smallest. Then, with the increased glucose content in feeding, the intensity of the diffraction peaks increased gradually until the maximum value appeared as glucose content in feeding being 2%, which indicated that the crystallinity of sc-PLAG was increasing. However, if glucose content in feeding continued to increase, the diffraction peak intensity of sc-PLAG decreased gradually; that is, the crystallinity of sc-PLAG decreased with the increase of glucose content in feeding. The XRD results were consistent with those of DSC, which showed that the comprehensive effects of the *M*_w_ and *Ɖ* of PDLAG, stereocomplexation, amphiphilicity, and the interaction between glucose residues and l-lactic and d-lactic units would induce the change of crystallinity of sc-PLAG with the glucose content in feeding.

### 3.4. FI-IR Analysis of the Effect of Glucose Groups on sc-PLAG

**Figure 4:**
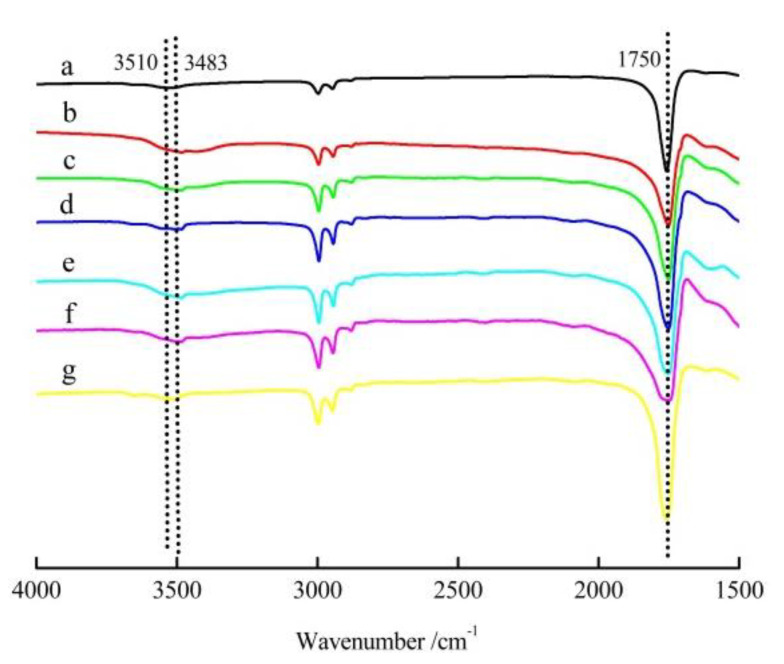
FT-IR spectra of PLLA, PDLAG-5%, and sc-PLAG samples in carbonyl region and hydrogen-bond region, a. PDLAG-5%; b. sc-PLAG-5%; c. sc-PLAG-4%; d. sc-PLAG-2%; e. sc-PLAG-1%; f. sc-PLAG-0.5%; g. PLLA.

In order to further investigate the role of glucose groups in sc-PLAG, the infrared spectroscopy was used to analyze the interaction of glucose groups with lactic units in sc-PLAG. Figure 4 shows the infrared spectra of PLLA, PDLAG-5%, and sc-PLGA samples in the carbonyl bond and hydrogen bond regions and crystalline regions. In Figure 4, the FT-IR spectrum of PLLA presented a relatively wide peak at 3483 cm^−1^ due to the stretch vibration of both free and hydrogen-bonded –OH groups and the strongest peak at 1750 cm^−1^ due to the stretch vibration of ester carbonyl C=O [6,38,39]. The FT-IR spectrum of PDLAG-5% resembled that of PLLA, while the wider peak of –OH groups moved to 3510 cm^−1^, and the C=O peak slightly redshifted with increasing peak intensity, which could result from the existence of glucose groups and its interaction with lactic units. When PLLA was blended with equimolar PDLAG to form SC structure, the IR spectra of sc-PLAG samples displayed that the peaks at 3510 cm^−1^ gradually widened and strengthened and slightly moved to low wavenumber with the increase of glucose content in feeding. These changes of -OH peaks could be attributed to the comprehensive actions of -OH groups existing in l-lactic units, d-lactic units, and glucose groups and their interactions growing with the increase of glucose content in feeding [40]. Meanwhile, compared with PLLA, the C=O peak of sc-PLAG samples slightly shifted to lower wavenumber with decreasing intensity along with the increase of glucose content in feeding, which might be due to the interaction between C=O groups with –OH groups of glucose. The FT-IR results showed that there was the interaction of glucose with lactic acid units, which was already demonstrated in DSC and XRD.

### 3.5. TGA Analysis of the Thermal Properties of sc-PLAG

**Figure 5:**
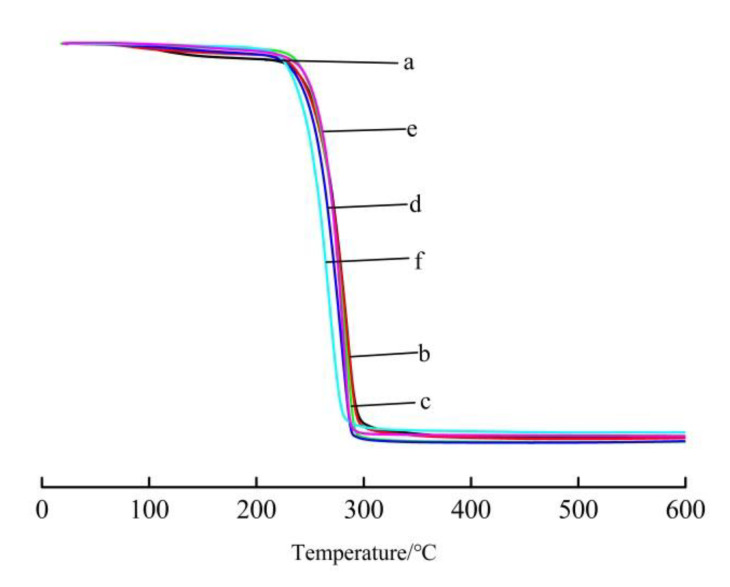
TGA curves of PLLA and sc-PDLAG samples with different glucose content in feeding; glucose content in the feeding of samples (%): a. 5%; b. 4%; c. 2%; d. 1%; e. 0.5%; f. PLLA.

Thermogravimetric analysis is also effective to evaluate the thermal properties of polymer materials. Figure 5 shows the TGA curves of PLLA and sc-PLAG samples. The beginning degradation temperature *T*_b_ (at 5 wt% mass loss), the maximum degradation temperature *T*_max_ (at 50 wt% mass loss), the final degradation temperature *T*_f_ (at 95 wt% mass loss) [41], and the carbon residue ratio *W*_r_ at 600 °C [26] of each sample were obtained from the analysis of Figure 5, and the results are listed in Table 3.

It could be reported from Figure 5 that PLLA and all sc-PLAG samples experienced single-step decomposition and had similar thermal degradation behaviors, namely, the thermal degradation process was divided into three stages. At the first stage, the weight loss of samples was less than 5% within 220 °C, which was due to the volatilization of a small amount of water left in samples and the pyrolysis of impurities. The second stage was at a temperature of 220–300 °C, which was the main pyrolysis stage, mainly due to lactic units and glucose groups, and up to 90 wt% of samples degraded. At the third stage, with the temperature higher than 300 °C, the pyrolysis of the samples reached the platform stage, and the *W*_r_ of PLLA and sc-PLAG samples was basically the same. PDLAG samples had thermal degradation behaviors similar to those of PLLA and sc-PLAG, which are listed in Table 3. Glucose groups possessed good thermal stability, and the increasing glucose proportion also increased the interaction of glucose groups with lactic units, which was helpful in retarding the pyrolysis process and improve the thermal stability of samples. However, glucose units could participate in the crystallization of PLA, decrease crystal perfection, and reduce thermal stability. The high *M*_w_, perfect crystal structure, and high distribution of glucose units on the surface would improve the thermal stability of PDLAG and sc-PLAG samples. The *T*_max_ of all PDLAG was lower than that of PLLA (its *T*_max_ being about 274 °C), according to Table 3, which indicated that glucose units might decrease crystal perfection and accelerate the thermal degradation. PDLAG-5% had the highest *M*_w_ and likely more low-*M*_w_ fractions moving to the surface easily, which resulted in the highest *T*_max_ of all PDLAG. On the contrary, PDLAG-2% had the lowest *T*_max_ because of its lowest *M*_w_ and highest glucose units, which could decrease crystal perfection. Moreover, the *T*_max_ of sc-PLAG samples was higher than that of the corresponding PDLAG, mainly due to its SC structure. On account of the comprehensive effects of the above factors, the *T*_max_ of sc-PLAG-0.5% was about 262 °C, then the *T*_max_ of sc-PLAG samples increased steadily with the increase of glucose content in feeding until the glucose content in feeding was 5%. Compared to PLLA-glucose blends [26] and PLA with different processing methods [41], the *T*_b_, *T*_max_, and *T*_f_ of all sc-PLAG were lower than those of PLLA-glucose blends and the PLA materials, which indicated that the copolymerization of poly(lactic acid) and glucose and stereocomplex of PLA was more able to promote thermal degradation of PLLA.

### 3.6. POM Analysis of Crystal Morphology

**Figure 6:**
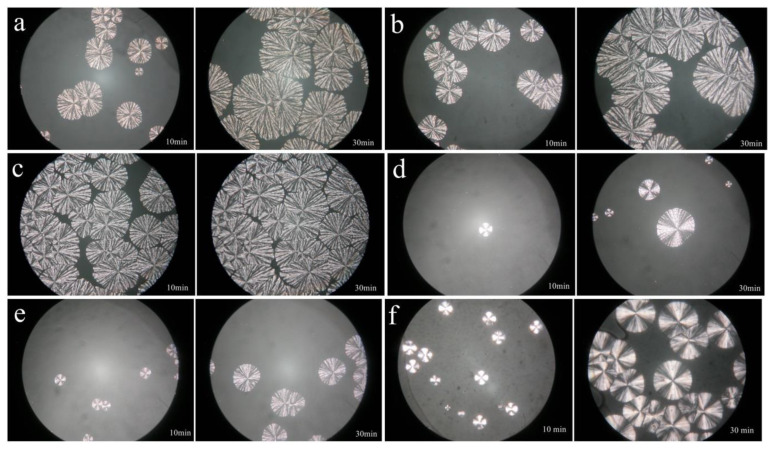
Polarizing microscope images of PLLA and sc-PLAG samples (X200); the glucose content in the feeding of samples: (**a**) 5%; (**b**) 4%; (**c**) 2%; (**d**) 1%; (**e**) 0.5%; (**f**) PLLA.

The crystallization process and the morphology of PLLA and sc-PLAG were observed by polarized optical microscope (POM). PLLA and sc-PLAG samples were melted at 165 °C and 230 °C, respectively, for 5 min, cooled to a certain temperature (PLLA at 120 °C, sc-PLAG at 190 °C) at a rate of 10 °C/min, and then maintained at a constant temperature. We could estimate the effects of stereocomplexation, chain amphiphilicity, and the interaction of glucose groups with lactic units on the properties of sc-PLAG by studying the crystallization process and morphology of sc-PLAG [42]. Figure 6 shows the POM images of PLLA and sc-PLAG samples with different glucose content in feeding, showing the process of spherulite growth from 10 min to 30 min. The crystallization of PLLA is shown in Figure 6f, with existing regular and uniform-sized spherulites and obvious black cross extinction phenomena. The size of spherulites gradually increased as the extension of time, and spherulites would contact each other and deform. The crystallization of sc-PLAG-0.5% is shown in Figure 6e, with existing regular and uniform-sized spherulites and obvious black cross extinction phenomenon, but the size and amount of spherulites in this sample was smaller than those of PLLA during the same period. The spherulites size of sc-PLAG increased gradually along with the increase of glucose content in feeding and reached the maximum as glucose content in feeding being 2% (Figure 6c), meaning the increase of crystallization rate. However, when glucose content in feeding continued increasing, the size of the spherulites of sc-PLAG gradually decreased, meaning the decrease of crystallization rate. The sc-PLAG samples with glucose content in feeding beyond 2% possessed the crystallization rate faster than that of PLLA. The black cross extinction phenomenon of spherulites became invisible at the same time, meaning the less ordered arrangement of spherulites. These variations of spherulites were due to the combined effect of PDLAG motion, the stereocomplexation, the amphiphilicity, and the interaction of glucose residues with lactic units, as mentioned above [3,31].

The results of POM showed that a proper amount of glucose groups would play the role of heterogeneous nucleation agent and improve the crystallization rate of PLA stereocomplex, while a small amount or an excess glucose group could destroy the ordered arrangement of lactic acid units to reduce the regularity of SC.

### 3.7. TEM and SEM Analysis of Sample Morphology

**Figure 7:**
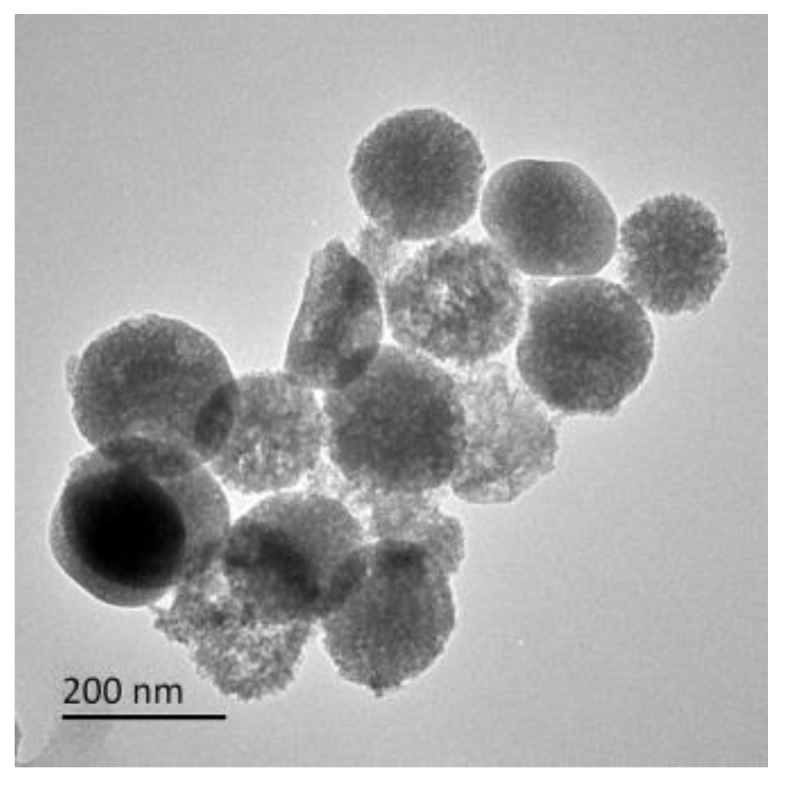
The TEM photograph of sc-PLAG-5%.

Figure 7 is the TEM photograph of sc-PLAG-5%, and other sc-PLAG samples also possessed similar morphology. The microspheres with a diameter of about 200 nm and the aggregation of microspheres could be observed [43]. In sc-PLAG, the formation of microspheres could be induced by the stereocomplexation of enantiomeric PLA units and the amphiphilicity due to glucose residues and lactic acid units, which indicated that the hydrophobic PLA units likely spontaneously gathered in the center of microspheres to form SC structure, while the hydrophilic glucose groups distributed on the surface of the microspheres. The self-assembly could be promoted by the stereocomplexation of enantiomeric PLA units, which would further improve the hydrophilicity of the surface of sc-PLAG samples.

**Figure 8:**
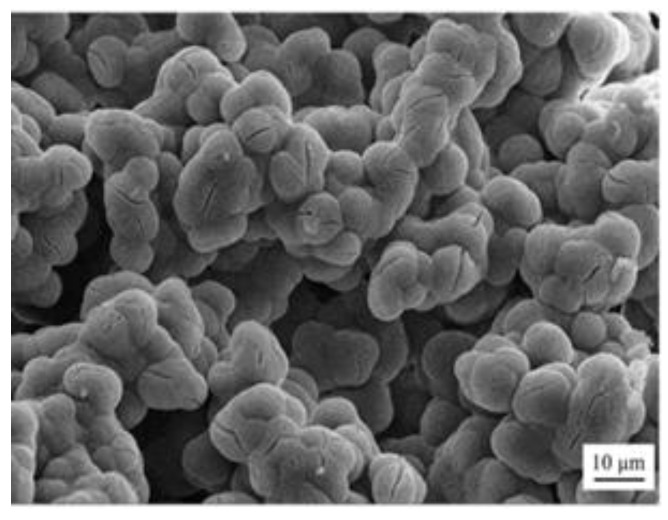
The SEM micrograph of the section of sc-PLAG-4%.

Figure 8 is the scanning electron micrograph of sc-PLAG-4%, and other sc-PLAG samples also possessed similar morphology. Sc-PLAG possessed a porous multilayer structure, which consisted of microsphere structure due to the self-assembly and the stereocomplexation. The porous layer structure might be caused by the pores generated by solvent evaporation during the preparation and drying process, which would improve the adsorption of water. The results of TEM and SEM indicated that sc-PLAG could form a microsphere and porous layer structure, which might provide the possibility to improve the hydrophilicity and degradation of PLA. The improvement of sc-PLAG hydrophilicity would also be further proved by the contact angles analysis below.

### 3.8. Hydrophilic Analysis of sc-PLAG

**Figure 9:**
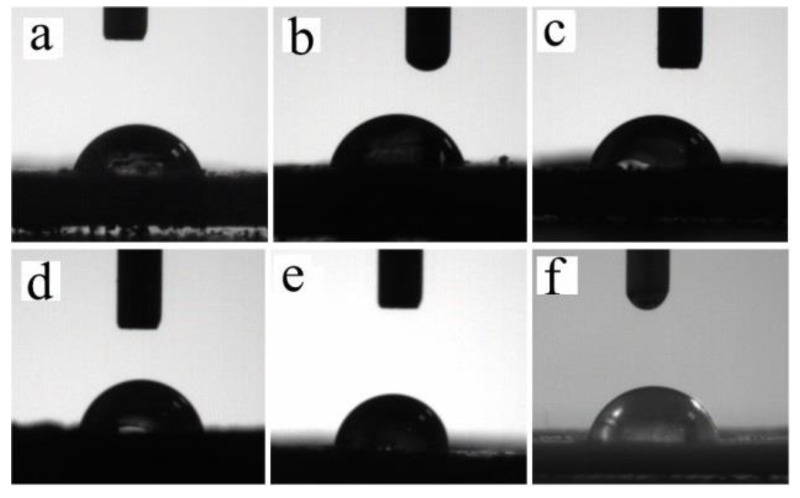
Water contact angles photographs of PLLA and sc-PLAG samples; the glucose content in the feeding of samples: (**a**) 5%; (**b**) 4%; (**c**) 2%; (**d**) 1%; (**e**) 0.5%; (**f**) PLLA.

In order to study the hydrophilicity of sc-PLAG samples, the water contact angles of sc-PLAG with different glucose content in feeding were tested [44]. Figure 9 shows the contact angle photographs of sc-PLAG blends and PLLA, respectively, and Table 4 shows the contact angle parameters of PLLA and sc-PLAG with different glucose content in feeding. The contact angles of all PLAG samples were less than that of PLLA, which meant that glucose could improve the hydrophilicity of PLA materials. The glucose residues in PDLAG with low *M*_w_ and wide *Ɖ* more easily moved to the surface and increased the hydrophilicity; thus, the contact angles of PLAG varied with glucose contents. The contact angles of all sc-PLAG were less than those of PDLAG because the interaction of PDLAG with PLLA could hinder PDLAG from moving to the surface. According to Figure 9 and Table 4, the contact angle of PLLA was 85°, indicating the poor hydrophilicity of PLLA. The contact angles of sc-PLAG decreased significantly with the increase of glucose contents in feeding by impacting the *M*_w_ and *Ɖ* of PDLAG samples. PDLAG-5% with the highest *Ɖ* perhaps contained more low-*M*_w_ components. As for sc-PLAG, multi-armed PDLAG interacted with linear PLLA, and the low-*M*_w_ glucose groups would tend to move to the surface of sc-PDLAG and improve its hydrophilicity. Thus, the contact angle of sc-PLAG-5% decreased to 77.5°. The result of contact angles testified that the addition of hydrophilic glucose was able to improve the hydrophilicity of PLA blends because the glucose groups were mainly located on the sc-PLAG surface due to the amphiphilicity of glucose residues and lactic units, which was consistent with SEM and TEM results.

To further explore the influence of stereocomplex and hydrophilic glucose groups on the water absorption performance of PLA materials [45], we tested and calculated the water absorption ratios of samples, and the results are listed in Table 5.

Table 5 shows the water absorption ratios of PLLA, PDLAG, and sc-PLAG with different glucose contents in feeding. The water absorption ratios of PDLAG samples were more than that of PLLA and increased with glucose contents. The water absorption ratios of sc-PLAG samples increased significantly compared with that of PLLA and more than that of the corresponding PDLAG. The water absorption ratio sc-PLAG-2% reached to the maximum, other than sc-PLAG-4% and sc-PLAG-5%, indicating that the water retention property of sc-PLAG did not merely depend on glucose contents in feeding. The water absorption ratios of sc-PLAG samples demonstrated that the addition of glucose significantly improved the hydrophilic property of PLA materials because of the porous structure and glucose groups mainly located on the surface of sc-PLAG. The result of water absorption showed that the addition of hydrophilic glucose was able to improve the hydrophilicity of PLA blends, which was consistent with SEM and contact angle analysis.

## 4. Conclusions

We successfully prepared sc-PLAG from PLLA and its enantiomeric copolymer PDLAG by direct polymerization and solution blending. PDLAG was multi-armed, and partial OH groups of glucose residues in PDLAG might remain unreacted. The glucose content in feeding obviously affected the *M*_w_, dispersity (*Ɖ*), and glucose proportion in the chain of PDLAG samples. The glucose content in feeding would play an important role in sc-PLAG through its influence on the *M*_w_, glucose proportion, and *Ɖ* of PDLAG. The results from DSC, XRD, and POM showed that only SC of sc-PLAG was formed when PLLA blended with equimolar PDLAG. The *T*_g_, *T*_m_, crystallinity, crystallization rate, and water absorption ratio presented similar changes with the increase of glucose content in feeding in sc-PLAG. That is, these performances of sc-PLAG gradually increased with the increase of glucose content in feeding until the maximum as glucose content in feeding being 2%. When glucose content in feeding continued increasing, these performances gradually decreased. The influence factors of the crystallization of sc-PLAG included the *M*_w_ and *Ɖ* of PDLAG, stereocomplexation of enantiomeric PLA units, the amphiphilicity due to glucose residues and lactic acid units, and the interaction between glucose residues and l-lactic and d-lactic acid units. The comprehensive actions of these factors resulted in different trends in these properties of sc-PLAG. Meanwhile, sc-PLAG possessed regular microsphere structure with glucose as the surface phase and SC of lactic units as the bulk phase from the results of TEM, SEM, contact angle analysis, and water absorption of sc-PLAG. Thus, poly(lactic acid) stereocomplex containing glucose groups could effectively improve the crystallinity, hydrophilicity, and thermal stability of PLA materials, providing a promising prospect in its application in the field of biomedicine, such as drug delivery system and tissue regeneration, etc.

## Figures and Tables

**Table 1:** The *M*_n_, *M*_w_, and dispersity (*Ɖ*) of PLLA and PDLAG by GPC.

Sample	Glucose Content in Feeding/%	Glucose Proportion in PDLAG/%	*M*_n_/g⋅mol^−1^	*M*_w_/g⋅mol^−1^	*Ɖ*
PDLAG-5%	5.0	1.64	10,700	20,600	1.93
PDLAG-4%	4.0	2.04	8560	16,400	1.92
PDLAG-2%	2.0	2.13	8200	15,600	1.90
PDLAG−1%	1.0	1.96	8900	16,500	1.85
PDLAG-0.5%	0.5	1.90	9230	16,800	1.83
PLLA	0	0	8760	17,000	1.95

*M*_n_: Number average molecular weight. *M*_w_: Weight average molecular weight.

**Table 2:** Thermal properties and crystallinity of PLLA, PDLAG, and sc-PLAG samples.

Sample	Glucose Content in Feeding/%	*T*_g_/°C	*T*_m_/°C	Crystallinity/%
PDLAG-5%	5.0	52.9	133.1	25.1
PDLAG-4%	4.0	51.3	131.7	24.8
PDLAG-2%	2.0	51.8	130.8	30.3
PDLAG-1%	1.0	57.2	132.6	34.8
PDLAG-0.5%	0.5	60.6	140.8	42.1
sc-PLAG-5%	5.0	51.8	203.5	38.3
sc-PLAG-4%	4.0	57.3	203.1	39.8
sc-PLAG-2%	2.0	65.4	208.1	48.8
sc-PLAG-1%	1.0	66.0	206.5	42.9
sc-PLAG-0.5%	0.5	53.9	205.6	35.9
PLLA	0	65.7	147.9	48.4

**Table 3:** Characteristic temperatures of PLLA, PLLAG, and sc-PLAG samples determined by TGA.

Sample	Glucose Content in Feeding/%	*T*_b_/°C	*T*_max_/°C	*T*_f_/%	*W*_r_/%
sc-PLAG-5%	5.0	227.77	276.48	306.47	2.50
sc-PLAG-4%	4.0	231.57	275.98	300.11	2.20
sc-PLAG-2%	2.0	239.21	274.60	291.05	1.32
sc-PLAG-1%	1.0	228.13	270.22	287.58	1.41
sc-PLAG-0.5%	0.5	227.17	262.89	299.25	3.60
PDLAG-5%	5.0	237.32	274.16	--	5.98
PDLAG-4%	4.0	224.44	263.03	335.69	4.62
PDLAG-2%	2.0	211.06	250.52	266.74	1.46
PDLAG-1%	1.0	213.35	254.17	293.89	4.34
PDLAG-0.5%	0.5	220.06	258.15	301.26	4.78
PLLA	0	237.59	274.37	288.75	2.71

**Table 4:** Water contact angles of PLLA and sc-PLAG samples.

Sample	Glucose Content/%	Contact Angle/°
sc-PLAG-5%	5.0	77.5
sc-PLAG-4%	4.0	78.5
sc-PLAG-2%	2.0	81.6
sc-PLAG-1%	1.0	82.1
sc-PLAG-0.5%	0.5	82.9
PDLAG-5%	5.0	70.5
PDLAG-4%	4.0	72.5
PDLAG-2%	2.0	66.5
PDLAG-1%	1.0	72.5
PDLAG-0.5%	0.5	76.0
PLLA	0	85.0

**Table 5:** Hydroscopic property of PLLA and sc-PLAG samples.

Sample	Glucose Content/%	Water Absorption Ratio/%
sc-PLAG-5%	5.0	9.7
sc-PLAG-4%	4.0	9.4
sc-PLAG-2%	2.0	11.7
sc-PLAG-1%	1.0	8.3
sc-PLAG-0.5%	0.5	7.5
PDLAG-5%	5.0	9.0
PDLAG-4%	4.0	8.7
PDLAG-2%	2.0	8.3
PDLAG-1%	1.0	7.8
PDLAG-0.5%	0.5	7.5
PLLA	0	2.5
